# Aged Cattle Brain Displays Alzheimer's Disease-Like Pathology and Promotes Brain Amyloidosis in a Transgenic Animal Model

**DOI:** 10.3389/fnagi.2021.815361

**Published:** 2022-01-31

**Authors:** Ines Moreno-Gonzalez, George Edwards, Rodrigo Morales, Claudia Duran-Aniotz, Gabriel Escobedo, Mercedes Marquez, Marti Pumarola, Claudio Soto

**Affiliations:** ^1^Department of Neurology, Mitchell Center for Alzheimer's Disease and Related Brain Disorders, University of Texas Health Science Center at Houston, Houston, TX, United States; ^2^Departamento Biología Celular, Genética y Fisiología, Instituto de Investigacion Biomedica de Malaga-IBIMA, Universidad de Malaga, Malaga, Spain; ^3^Center for Biomedical Research on Neurodegenerative Diseases (CIBERNED), Madrid, Spain; ^4^Centro Integrativo de Biologia y Quimica Aplicada (CIBQA), Universidad Bernardo O'Higgins, Santiago, Chile; ^5^Center for Social and Cognitive Neuroscience (CSCN), School of Psychology, Universidad Adolfo Ibáñez, Santiago, Chile; ^6^Latin American Institute for Brain Health (BrainLat), Universidad Adolfo Ibanez, Santiago, Chile; ^7^Department of Animal Medicine and Surgery, Veterinary Faculty, Animal Tissue Bank of Catalunya (BTAC), Universitat Autònoma de Barcelona, Bellaterra (Cerdanyola del Valles), Barcelona, Spain; ^8^Networking Research Center on Bioengineering, Biomaterials and Nanomedicine (CIBER-BBN), Universitat Autonoma de Barcelona, Bellaterra (Cerdanyola del Valles), Barcelona, Spain

**Keywords:** amyloid, prions, Alzheimer's disease, spreading, protein misfolding, seeding, cattle

## Abstract

Alzheimer's disease (AD) is one of the leading causes of dementia in late life. Although the cause of AD neurodegenerative changes is not fully understood, extensive evidence suggests that the misfolding, aggregation and cerebral accumulation of amyloid beta (Aβ) and tau proteins are hallmark events. Recent reports have shown that protein misfolding and aggregation can be induced by administration of small quantities of preformed aggregates, following a similar principle by which prion diseases can be transmitted by infection. In the past few years, many of the typical properties that characterize prions as infectious agents were also shown in Aβ aggregates. Interestingly, prion diseases affect not only humans, but also various species of mammals, and it has been demonstrated that infectious prions present in animal tissues, particularly cattle affected by bovine spongiform encephalopathy (BSE), can infect humans. It has been reported that protein deposits resembling Aβ amyloid plaques are present in the brain of several aged non-human mammals, including monkeys, bears, dogs, and cheetahs. In this study, we investigated the presence of Aβ aggregates in the brain of aged cattle, their similarities with the protein deposits observed in AD patients, and their capability to promote AD pathological features when intracerebrally inoculated into transgenic animal models of AD. Our data show that aged cattle can develop AD-like neuropathological abnormalities, including amyloid plaques, as studied histologically. Importantly, cow-derived aggregates accelerate Aβ amyloid deposition in the brain of AD transgenic animals. Surprisingly, the rate of induction produced by administration of the cattle material was substantially higher than induction produced by injection of similar amounts of human AD material. Our findings demonstrate that cows develop seeding-competent Aβ aggregates, similarly as observed in AD patients.

## Introduction

Alzheimer's disease (AD) is the most common form of dementia among elderly people and one of leading public health problems in developed countries. AD involves progressive brain atrophy, neuronal death, synaptic dysfunction, astrogliosis and the accumulation of protein aggregates in the form of amyloid-beta (Aβ) deposits, and tau neurofibrillary tangles. Although the etiology of AD is not yet clear, extensive evidence suggests that the central pathological event is the misfolding, aggregation and brain deposition of Aβ and tau (Soto, 2003; Huang and Mucke, 2012; Masters and Selkoe, 2012). Amyloid accumulates as senile plaques and diffuse deposits in the brain parenchyma and around cerebral blood vessels walls termed cerebral amyloid angiopathy (CAA) (Gomez-Isla et al., 2008).

In addition to AD, various other protein misfolding diseases (PMDs) are thought to be caused by accumulation of misfolded aggregated proteins in various tissues, including highly prevalent illnesses such as Parkinson's disease (PD), type 2 diabetes and more than 20 other diseases (Chiti et al., 2006; Soto, 2012). Among the latter, prion diseases are quite intriguing because they are transmissible by infection through a proteinaceous infectious agent known as prion (Prusiner, 1998). The molecular mechanism responsible for prion infectivity depends on the ability of the misfolded prion protein aggregates to act as seeds, inducing the templated conversion of natively folded prion protein into the developing aggregates (Soto, 2012). In this manner, the pathological protein progressively grows by recruiting more and more of the normal protein. This process is often referred as seeding/nucleation polymerization. Importantly, protein aggregation of all proteins involved in PMDs follows the seeding-nucleation mechanism (Caughey and Lansbury, 2003; Soto, 2012). The similarities between prion replication and amyloid formation, and the intrinsic ability of aggregated seeds to self-propagate the polymerization process led us and others to hypothesize over 10 years ago that misfolded aggregates associated to other PMDs can spread by the prion principle (Soto et al., 2006; Walker et al., 2006). Remarkably, a series of exciting recent reports have demonstrated that several PMDs can be experimentally transmitted by a prion-like mechanism in various cellular and animal models of diverse diseases (Prusiner, 2012; Walker and Jucker, 2015). Over the past decade, many of the hallmark properties of prions as infectious agents have been shown to be shared by several of the prion-like misfolded proteins. For the specific case of Aβ, studies from us and other groups have shown that inoculation of transgenic mouse models of amyloidosis with tissue homogenates from patients affected by AD results in induction or acceleration of amyloid pathology in the recipient animals (Kane et al., 2000; Meyer-Luehmann et al., 2006; Morales et al., 2011; Watts et al., 2011). Moreover, in animals not genetically programmed to develop the disease spontaneously, inoculation with AD brain homogenates leads to a completely *de novo* disease, more akin to infectious prions (Morales et al., 2011; Rosen et al., 2011). Importantly, pathological induction can be reduced by depleting the inoculum of Aβ aggregates (Meyer-Luehmann et al., 2006; Duran-Aniotz et al., 2014). Even more strikingly, efficient induction has been observed by the addition of misfolded protein aggregates prepared *in vitro* using synthetic Aβ (Stöhr et al., 2012). Accumulation of Aβ aggregates can be promoted by inoculation of very small amounts of aggregated seeds (Fritschi et al., 2014; Morales et al., 2015a) and titration experiments have shown that the rate of induction is proportional to the amount of seeds inoculated. Finally, disease transmission has been observed even when seeds were administered systemically (Eisele et al., 2010).

The findings described above suggest that Aβ and other misfolded protein aggregates can indeed behave as prions. Still, the main controversy is whether other misfolded proteins can act as infectious agents to transmit the disease among individuals under natural conditions (Fernández-Borges et al., 2013; Irwin et al., 2013; Beekes et al., 2014; Collinge, 2016). Aside from this important point, another aspect that has not been explored is the possibility that protein aggregates accumulating in animals may initiate the disease in humans. Indeed, prion diseases affect not only humans, but also various species of mammals. The accumulation of Aβ aggregates has not been extensively analyzed in animals, however, it has been reported that AD aggregates are present in the brain of several aged non-human mammals, including monkeys, bears, dogs, and cheetahs (Moreno-Gonzalez and Soto, 2018). In bovine brains, Aβ has been as previously observed as granular aggregates, but not depositing in plaques (Costassa et al., 2016). In this study, we investigated the presence of AD-like pathology in aged cow brains and whether, in analogy to prion diseases, Aβ aggregates derived from cattle brain can induce Aβ misfolding and aggregation in a transgenic mice model of AD amyloidosis.

## Materials and Methods

### Cattle Samples

Cattle samples were obtained from the Animal Tissue Bank of Catalunya (BTAC), Department of Animal Medicine and Surgery, Veterinary Faculty, Universitat Autonoma de Barcelona, Bellaterra (Cerdanyola del Valles), Barcelona, Spain. Brain samples were obtained from slaughterhouses after the animals were sacrificed. Samples with a *post-mortem* time lower than 10 h were immersed in formol, processed and embedded in paraffin. As shown in Supplementary Figure 1, we received 63 samples from female cattle that were 13 to 23 years old. These animals were from 10 different breeds, including Charolais, Bruna of Pirineus, Pirinenca, Limousin, Montbeliard, Simmental, and crossbreeds. We also obtained ten samples of 10 month-old young crossbreed calves that were used as controls. Samples were obtained from the temporal area and contained the hippocampal area, entorhinal cortex, and part of the thalamus.

### Human Samples

AD brain hippocampal samples were acquired from the National Disease Research Interchange (USA). Informed consent was obtained for experimentation with human subjects. The Code of Ethics of the World Medical Association (Declaration of Helsinki) was followed to perform research on human samples and they were manipulated following the universal precautions for working with human samples and as directed by the Institutional Review Board of McGovern Medical School at The University of Texas Health Science Center at Houston.

### Brain Homogenate

Cattle and human brain tissue were homogenized at 10% w/v in PBS containing a cocktail of protease inhibitors for western blot and ELISA quantifications. For intracerebral inoculations, samples were homogenized at 40% w/v in the same solution and sterilized by the addition of 1% of antibiotic/antimycotic solution (Gibco) and γ-irradiated for 1 h.

### Western Blot

10% cattle brain homogenates were mixed with denaturing loading buffer (Invitrogen), heated for 10 min at 95 °C and fractionated in 4–12% NuPAGE gels (Invitrogen). Proteins were transferred to a nitrocellulose membrane (GE Healthcare), blocked with 10% milk, and incubated with rabbit anti-Aβ42 polyclonal antibody (Covance). After incubation with secondary antibody, Aβ42 was visualized by chemoluminescence using ECL plus (GE Healthcare) in a dark chamber (BioRad).

### ELISA

Mouse brain hemispheres were homogenized at 10% w/v in PBS containing a cocktail of protease inhibitors. Brain extracts were centrifuged at 32,600 rpm for 1 h at 4°C in an ultracentrifuge (Beckman-Coulter). The pellets were resuspended in 200 μL of 70% formic acid followed by sonication. Samples were centrifuged for 30 min in the same conditions and the supernatant was collected. This insoluble fraction was neutralized in 1M Tris buffer, pH 11. Brain levels of Aβ42 were measured using a human Aβ ELISA kit (Invitrogen) on an ELISA plate reader (EL800 BIO-TEK).

### Histology

Serial 10-μm-thick sections from all cattle, human, and mouse groups were processed for immunostaining. After blocking the endogenous peroxidase activity with 3% H_2_O_2_−10% methanol for 20 min, sections were incubated overnight at room temperature in mouse anti-Aβ 4G8 (1:1,000 Covance). Sections stained for Aβ were pretreated with 85% formic acid. Primary antibody was detected by incubating 1 h with goat anti-mouse HRP-linked secondary antibody and the peroxidase reaction was visualized using a DAB Kit (Vector) following the manufacturer's instructions. For counterstaining, sections were incubated in Harris hematoxylin for 1 min (Fisher). For Thioflavin-S (ThS) staining, tissue slices were incubated in ThS solution (0.025% in 50% ethanol) for 8 min. Finally, all sections were dehydrated in graded ethanol, cleared in xylene, cover-slipped with DPX mounting medium, and examined under a bright field/epifluorescent microscope (DMI6000B, Leica).

### Animals

Hemizygous APP/PS1 (B6C3-Tg APPswe, PSEN1dE9 85Dbo/J, The Jackson Laboratory) mice express human amyloid precursor protein (APP695swe) and a mutant human presenilin 1 (PS1-dE9) in a B6C3 background. These animals develop amyloid plaques and other AD-like features starting around 6 months of age (Jankowsky et al., 2004). Animals were housed in groups of up to five in individually ventilated cages under standard conditions (22°C, 12 h light–dark cycle) receiving food and water *ad libitum*. All animal experiments were carried out in accordance with the NIH regulations and approved by the committee of animal use for research at The University of Texas Health Science Center at Houston. Mice were sacrificed by CO_2_ inhalation and perfused transcardially with PBS. Brains were removed, post-fixed into fixative solution (10% neutral buffered formalin) and embedded in paraffin.

### Animal Inoculation

For intracerebral inoculation, 30–40 days-old APP/PS1 mice were injected with 10 μL of 40% (w/v) cattle or human brain homogenate in each hemisphere (*n* = 8 to 11 per group), without any purification or isolation. Briefly, mice were anesthetized using isoflurane. Skin was incised and a small hole was drilled in the skull and samples were injected into both hippocampi. At the end of the treatment, skin was closed using surgical suture. Animals were placed on a thermal pad until recovery and monitored daily for several days.

### Histological Quantification

Burden quantification was done through the lateromedial extent of the cortical and hippocampal areas in the sagittal plane, being the first section of each animal randomly collected. Quantification was assessed in every tenth section (with a distance of 100 μm among them), and four to six 10 μm sections were measured for each animal (*n* = 8–11 per group). Photomicrographs were taken by using a DFC310 FX Leica digital camera, imported into ImageJ 1.45s software (NIH) and converted to black and white images. Threshold intensity was manually set and kept constant, and the number of pixels was determined for 4G8 immunostained sections to quantify amyloid load in the hippocampal formation (CA1, CA2, CA3, and dentate gyrus) and several cortical areas (including motor, somatosensory, visual, frontal, parietal and retrosplenial cortex). Analysis for each was done by a single examiner blinded to sample identities.

### Statistical Analysis

Graphs are expressed as means ± standard error (s.e.m.). After confirming normal distribution with Skewness and Kurtosis statistic test, *T*-test or one way analysis of variance (ANOVA) followed by a *post-hoc* Tukey's multiple comparisons test were used to analyze differences among groups. Statistical analyses were performed using GraphPad Prism 5.0 software (GraphPad Software Inc). Statistical differences for all tests were considered significant at the *p* < 0.05 level.

## Results

**Figure 1:**
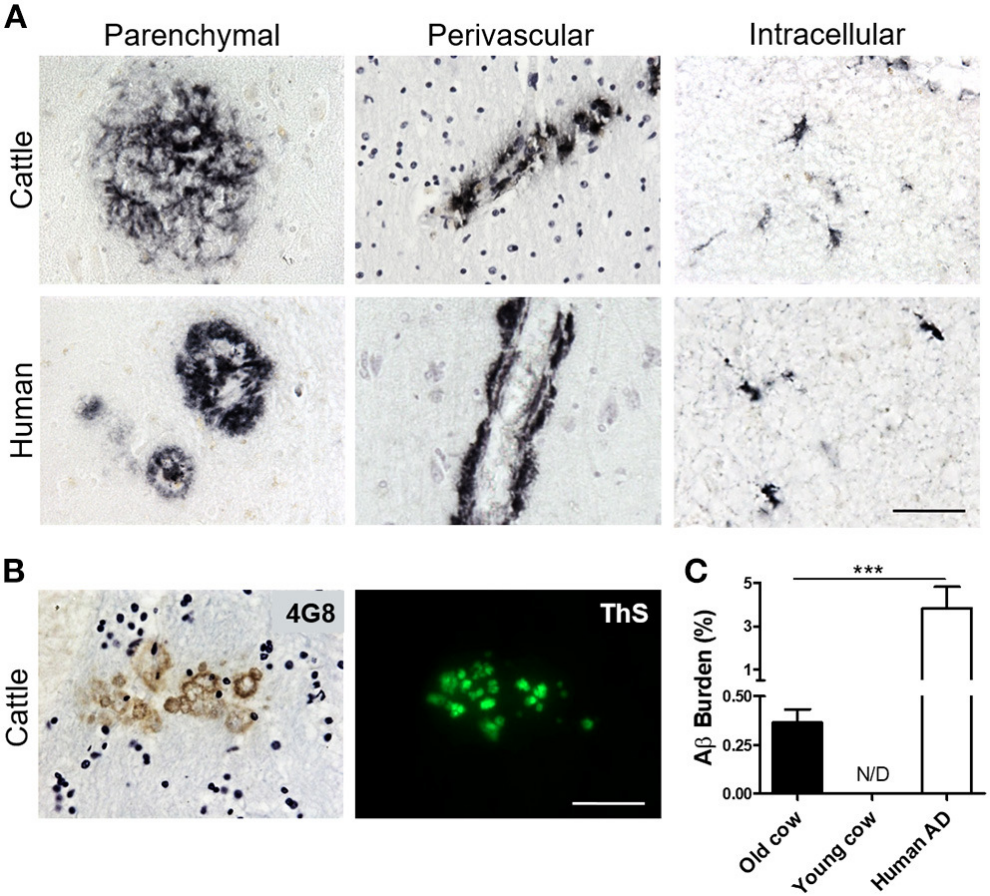
Accumulation of Aβ deposits in aged cattle brain. **(A)** Histological analysis of the brain of old cattle (top panels) showed the presence of diverse types of Aβ−immunoreactive lesions, including parenchymal amyloid plaques (left), perivascular deposits (center) and intracellular aggregates (right). These deposits were similar to those observed in patients affected by AD (bottom panels). Staining was done using the 4G8 anti-Aβ antibody in sections of the temporal area. **(B)** The parenchymal deposits were also ThS-positive when stained in the consecutive slide, showing that cattle aggregates also acquire fibrillar structure. **(C)** Histology data was analyzed to quantify the Aβ burden, defined as the percentage of the brain area occupied by Aβ−reactive deposits. Data represent the mean ± standard error of the mean and differences were analyzed by an unpaired student T-test (*p* < 0.001, *N* = 5–14). Scale bar in **(A)** and **(B)** corresponds to 50 μm. ***p < 0.001.

To investigate whether cattle develops AD-related abnormalities, we performed a detailed histological study for the presence of amyloid-like deposits in the brain of cows of different ages. The majority of the samples were from female cattle of the Bruna of Pirineus and various crossbreed (Supplementary Figure 1A). Old animal ages ranged from 13 to 23 years old (Supplementary Figure 1B). For this study, we analyzed the hippocampus, temporal cortex, and thalamus of more than 60 cows (Supplementary Figure 1C). For most of the specimens analyzed, we had access to both snap-frozen and formalin-fixed material. Positive and negative controls included human AD brains and 10 month-old calf brains, respectively. Paraffin embedded fixed tissue from all the specimens (cattle and human samples) were processed and stained for Aβ. The amino acid sequence of Aβ in both humans and cattle is exactly the same (Johnstone et al., 1991); therefore, we were able to use the same antibodies. After a complete histopathological analysis, we observed a scattered appearance of Aβ deposits in many slices of some aged cow brains (Figure 1A). Aβ deposits were detected in the cortex and hippocampus of various cows >13 years old, but not in any young animals. Cow amyloid deposits were reactive against human anti-Aβ antibody (4G8), depositing as mature plaques similar to those seen in human AD patients' brains (Figure 1A, left panels). We also observed perivascular deposits, which are highly reminiscent of cerebral amyloid angiopathy lesions commonly seen in human AD brains (Figure 1A, center panels). In addition, intracellular Aβ staining was observed in both cattle and human brains (Figure 1A, right panels). Most of the deposits observed in cattle brain corresponded to fibrillar amyloid aggregates, as confirmed by the positive staining of the plaques using thioflavin-S (ThS) in the consecutive slice (Figure 1B). In total, 14 out of 63 brains from cows older than 13 years of age displayed Aβ aggregates in the brain areas studied, although just nine of them presented Aβ plaques similar to those observed in humans (14.3%). To examine the relative amount of amyloid deposits observed in cattle brain and compare it with that present in patients affected by AD, we performed image analysis of the temporal area from 14 old cows exhibiting amyloid pathology and five patients with AD (Figure 1C). The results show that the brains of old cows have a substantial amount of amyloid deposits. Indeed, in the hippocampal region, the animals analyzed showed an amyloid burden of 0.37 ± 0.07%, which means that 0.37% of the brain area analyzed was occupied by amyloid. Nevertheless, this burden is significantly smaller (>10-fold lower) than the one estimated on the AD patients (3.82 ± 1.0%) analyzed (Figure 1C). We could not detect any Aβ aggregates in young cattle brain samples.

**Figure 2:**
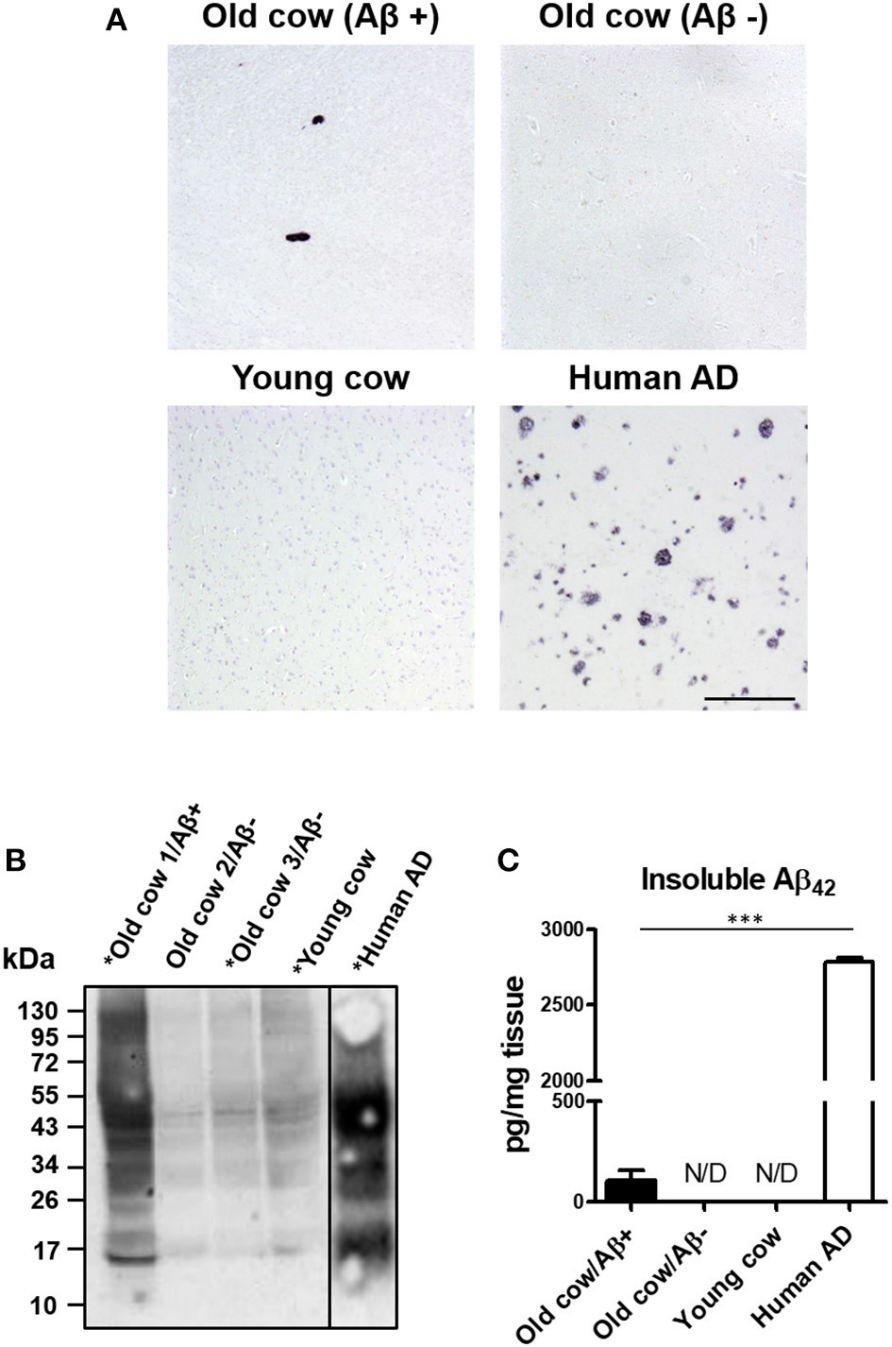
Histological and biochemical characterization of materials used for *in vivo* transmission experiments. **(A)** Representative pictures of cattle brains stained with the 4G8 anti-Aβ antibody that were utilized for *in vivo* inoculation, including young animals and old cows with (Aβ+) and without Aβ (Aβ−). The figure also shows the staining for the AD human brain utilized for these experiments as a positive control. **(B)** Aliquots of 10 μL from the total brain extracts from old and young cows were analyzed by western blot using a rabbit-Aβ42 polyclonal antibody. For comparison, the same amount of the AD brain extract was also included. Samples labeled with an asterisk (*) were used for the *in vivo* transmission studies described in Figure 3. A black line separating the lanes indicates gel splicing. **(C)** Quantification of insoluble Aβ42 aggregates by serial extraction, centrifugation and ELISA (see Methodology) expressed in pg per mg of brain tissue. Data represent the average ± standard error of the mean for three replicates. Differences on the levels between human AD and old cow (Aβ+) brains were evaluated by unpaired student T-test (***p < 0.001). Insoluble Aβ was not detected (N/D) in either the old cow (Aβ−) or the young cow brain extracts.

We and others have shown that inoculation of Aβ−rich brain homogenate from AD patients or transgenic mice is able to induce an early AD-like pathology in recipient transgenic animals (Meyer-Luehmann et al., 2006; Eisele et al., 2010; Morales et al., 2011). Knowing that the sequence of cattle Aβ peptide is identical to the human one, we wanted to test whether cattle brain harboring amyloid aggregates exacerbate AD pathology in susceptible mice. To that end, we intracerebrally (i.c.) injected amyloid-containing cow brain tissue extracts into a double transgenic (APP/PS1) mouse model of brain amyloidosis (Jankowsky et al., 2004) and analyzed the possible acceleration of the pathology. For the experiment, the following five groups (*n* = 8–11 per group) of animals were used: (i) the experimental group injected with 40% brain homogenate from an old cow containing amyloid deposits (Old cow/Aβ+); (ii) a group injected with the same amount of old cow brain homogenate in which amyloid deposition was not detected by histological or biochemical analysis (old cow/Aβ−); (iii) a control group injected with 40% brain extract from a young cow; (iv) a negative control consisting of untreated animals; and (v) a positive control in animals injected with human AD brain homogenate. Before treatment, the inocula for the different groups were thoroughly analyzed by histological and biochemical techniques to measure the presence and quantity of Aβ aggregates. Figure 2A shows the comparative histological staining of representative brain sections from the different subjects used for inoculation. No amyloid staining was seen in any of the slices analyzed from the young cow or the old cow/Aβ−. In contrast, the old cow/Aβ+ exhibited scattered appearance of 4G8 positive deposits reminiscent of amyloid plaques, similar in morphology to human AD brains (Figure 2A). Biochemical analysis of cattle brain homogenate by western blot demonstrated that the specimen selected for the experimental group (old cow1/Aβ+) presented a wide range of high molecular weight species reactive with an antibody that recognizes specifically human Aβ42 (Figure 2B). Only some of these bands were detectable and at a much lesser intensity in 10-month-old young cow or the old cows/Aβ−, used as age-matched controls. The samples indicated with an asterisk in Figure 2B were used for the transmissibility experiments described below. Finally, we measured the amount of Aβ42 in the insoluble fraction after extraction with formic acid in each of the inocula (Figure 2C). The results showed a concentration of insoluble Aβ42 of 31.72 pg/mg of tissue in the old cow/Aβ+. In contrast, no detectable insoluble Aβ42 was observed in the young cow or the old cow/Aβ−. It is important to highlight that even though the levels of insoluble Aβ42 in the old cow/Aβ+ brain were significantly higher than in young animals, they represent only around 1% of the levels detectable in human AD brain homogenate (2,788.78 pg/mg) (Figure 2C).

**Figure 3:**
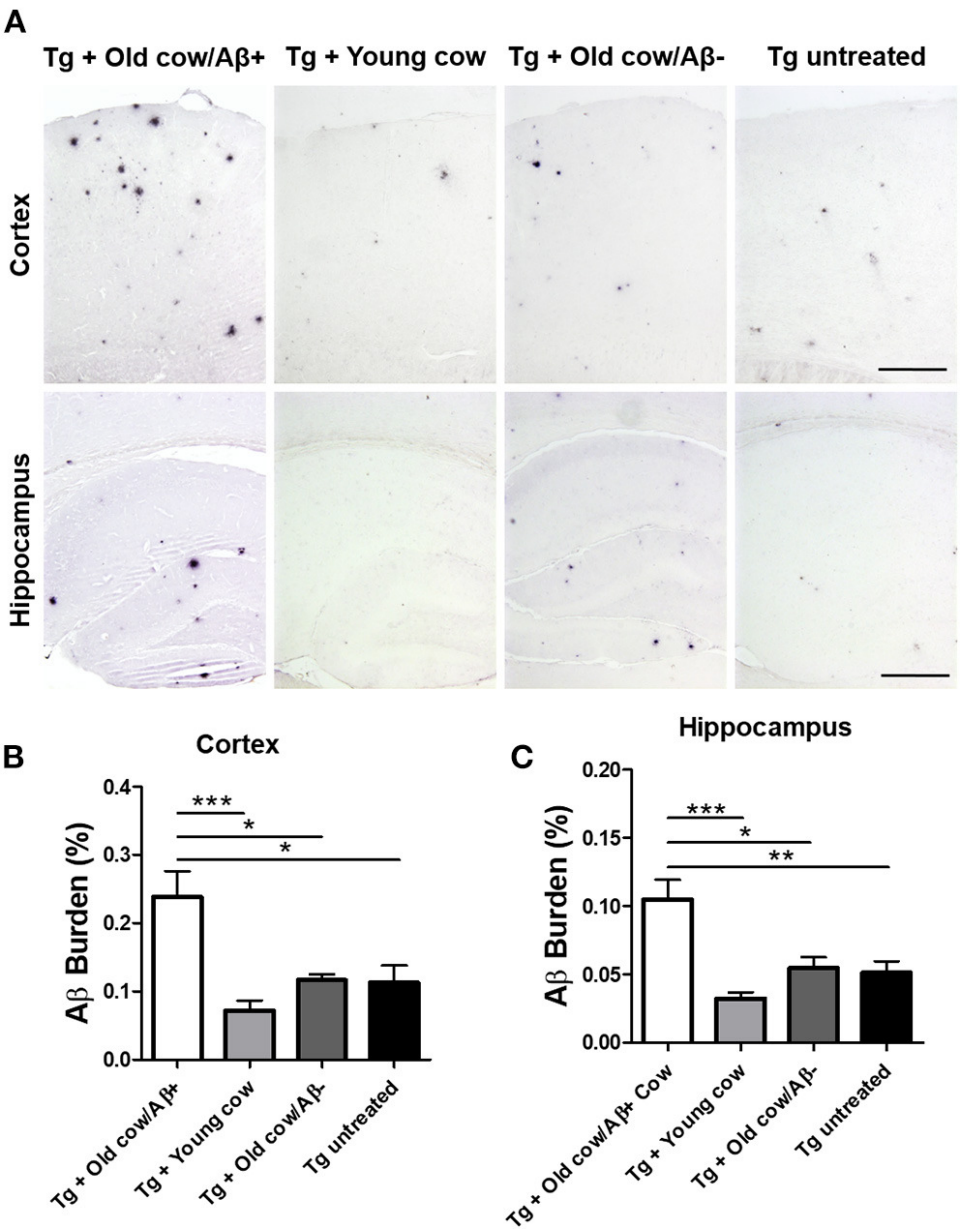
Acceleration of amyloid pathology in transgenic mice by injecting Aβ−rich cow brain extract. APP/PS1 double transgenic mice (30–40 days old) were injected intra-cerebrally (both hemispheres at the level of the hippocampus) with 10 μL of 40% (w/v) brain extracts of a young cow or old cows with (Aβ+) or without (Aβ−) Aβ. Control animals were left untreated. Animals were sacrificed at 6 months of age and their brains were fixed for histological analysis. **(A)** Representative pictures of 4G8 immunostaining of the cortex and hippocampal areas. Scale bar: 250 μm. Aβ burden was quantified by image analysis in the cortex **(B)** and the hippocampus **(C)**. Data corresponds to the average ± standard error of the mean of all animals analyzed in each group (*n* = 7–9). Differences were analyzed by one-way ANOVA followed by the Tukey's multiple comparison post-test. *p < 0.05, **p < 0.01, ***p < 0.001.

**Figure 4:**
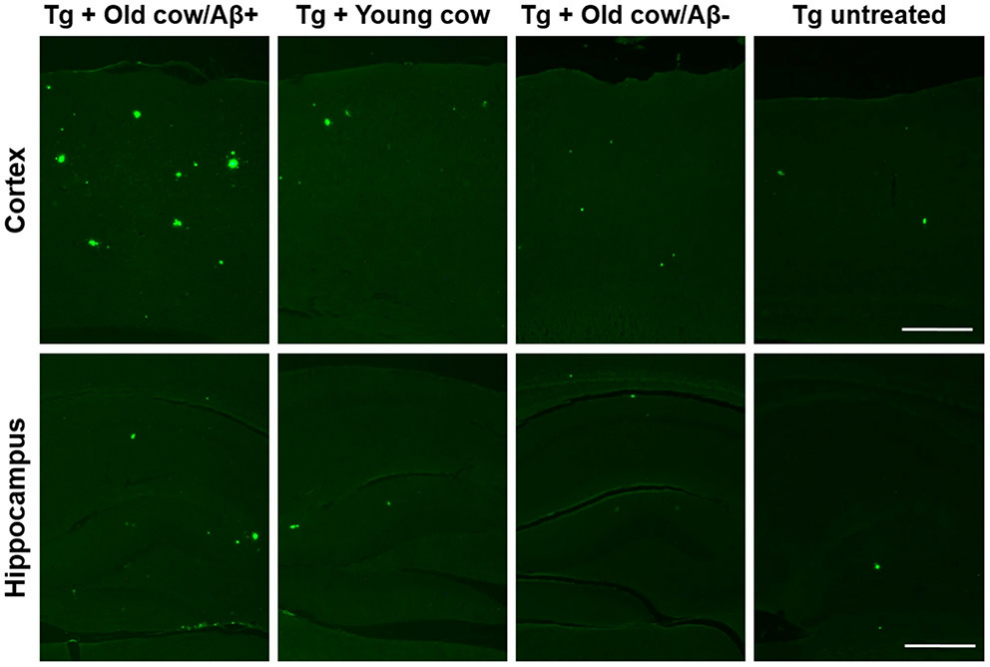
Aβ−rich cattle tissue induced high amounts of fibrillar plaques. Representative pictures of ThS staining in APP/PS1 transgenic mice inoculated with brain extracts from Aβ−containing old cattle, old cow with no detectable Aβ aggregates, and young cattle. For a negative control, we used transgenic mice left untreated. Scale bar: 250 μm.

To evaluate the ability of Aβ aggregates deposited in cattle brain to seed amyloid formation *in vivo*, double transgenic animals were injected i.c. at 30–40 days old with 10 μL of 40% amyloid-containing old cow brain homogenate into both hippocampal hemispheres through stereotactic surgery (Tg + Old cow/Aβ+). In addition, various groups of control animals were treated with the diverse inocula described above. Animals were sacrificed at 6 months-old, when they normally start developing plaques due to the transgenic expression of the mutant genes. 4G8 immunostaining showed that animals injected with amyloid-containing cattle brain homogenate displayed more amyloid plaques in the cortical area than the control groups (Figure 3A, top panels). When Aβ load was measured by image analysis, we could observe that there was a statistically significant increase in Aβ burden (stained area/total area analyzed) in the group injected with old cow/Aβ+. The increase was as much as 2-fold (0.24 ± 0.04 vs. 0.11 ± 0.03 %) compared to non-treated animals (Figure 3B). Analysis of Aβ deposition in the hippocampus, where the inoculation occurred, showed similar results (Figure 3A, bottom panels). Quantification of the hippocampal amyloid burden in experimental and control groups also showed that animals injected with Aβ−containing cattle brain homogenate doubled the regular amount of Aβ (0.11 ± 0.02 vs. 0.05 ± 0.01%) found in the hippocampus of APP/PS1 at this age (Figure 3C). Moreover, staining of fibrillar aggregates by ThS also showed that animals injected with Aβ−containing cow brain tissue displayed more ThS-positive plaques in both cortex and hippocampus than control groups (Figure 4). These results indicate that amyloid deposits present in cattle brain homogenate contain seeding-competent Aβ aggregates that are able to accelerate Aβ deposition when inoculated into a transgenic animal model of AD.

**Figure 5:**
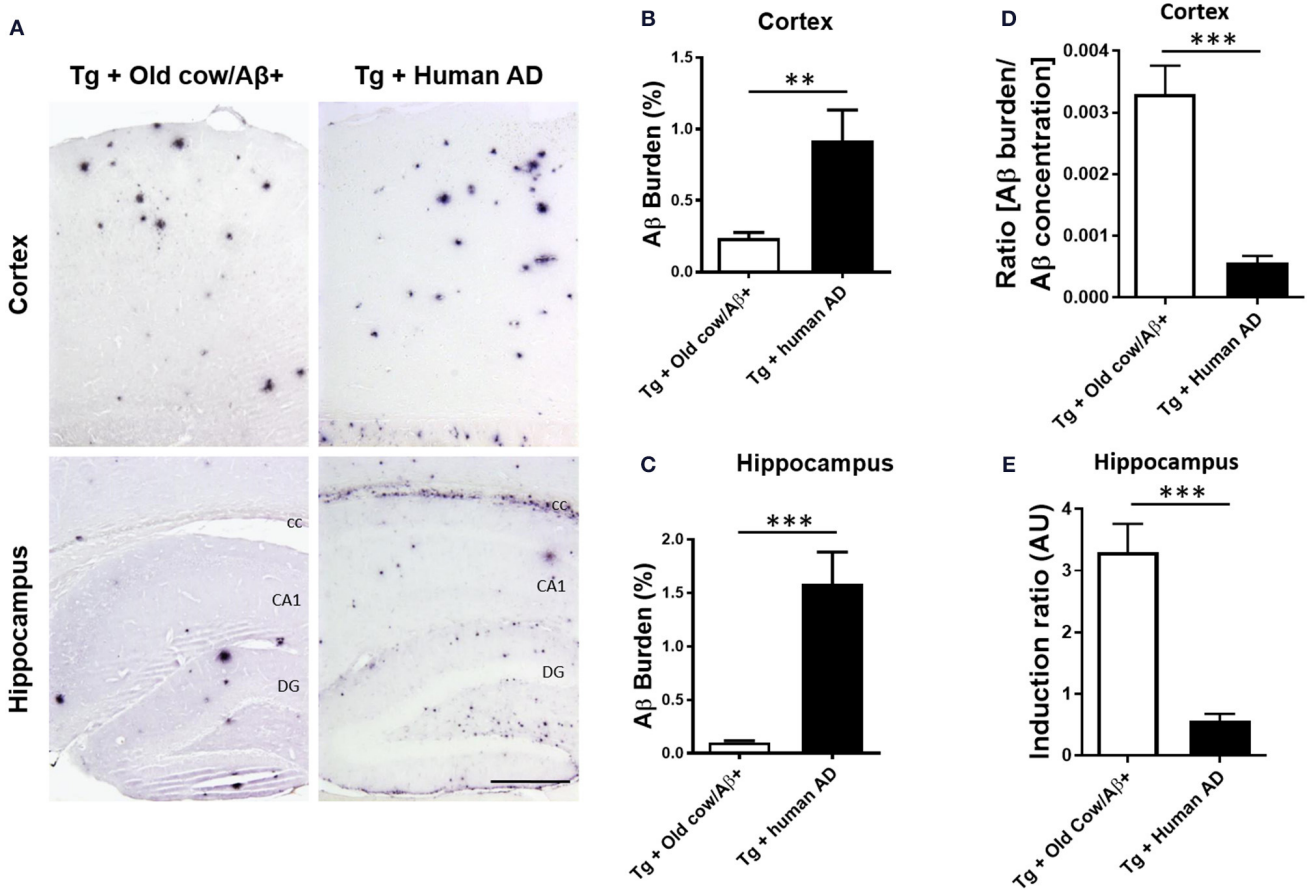
Comparison of amyloid induction by injecting Aβ− rich old cow and human AD brain extracts. **(A)** Representative pictures of 4G8 immunostaining in APP/PS1 transgenic mice inoculated with Aβ− containing old cattle brain extract or AD human brain homogenate. Pictures of the cortex and hippocampus regions are shown. Aβ burden in the cortex **(B)** and the hippocampus **(C)** was quantified by image analysis and expressed as the percentage of the Aβ immunoreactive area in relation to the total area analyzed. Data represents the average ± standard error of the animals analyzed (*n* = 7–9 per group). Since the amount of insoluble Aβ aggregates in the cow and human inoculum was substantially different, we estimated the induction ratio by dividing the Aβ burden (%) obtained in transgenic mice by the quantity of insoluble Aβ injected (pg/mg of brain) x 1,000 (arbitrary units, AU). Induction ratio was determined in the cortex **(D)** and the hippocampus **(E)** which was expressed as the average ± standard error of the mean. Data in panels B-E was analyzed by student T-test, **p < 0.01, ***p < 0.001. cc, corpus collosum; CA1, Cornu Ammonis area 1; DG, dentate gyrus.

To compare the seeding capability of cattle and human Aβ aggregates to induce amyloid deposition, APP/PS1 transgenic animals were also inoculated with human brain extracts from a patient affected by AD. The amyloid deposition pattern and burden was analyzed by immunohistochemistry in both groups. As shown in Figure 5A, the human inoculum was able to induce amyloid deposition to a substantially higher extent than the cattle inoculum in both cortex and hippocampus. In the cortical area, the human brain injection increases amyloid burden 4-fold more than the cattle inoculum (0.92 ± 0.22 vs. 0.24 ± 0.04%) (Figure 5B, *p* = 0.003 *T*-test), whereas in the hippocampus the increase was ~10-fold (1.59 ± 0.30 vs. 0.15 ± 0.2%) (Figure 5C, *p* < 0.0001 *T*-test). However, when the induction ratio (amyloid burden/concentration of insoluble Aβ42 injected) was calculated, the data indicates that cattle material promotes amyloid deposition >10-fold better than an equivalent quantity of human Aβ aggregates (Figures 5D,E). This surprising data suggests that although the cattle sample contains less concentration of aggregated Aβ, these structures appear to be more competent to seed amyloid deposition than human samples. In addition, the human aggregates produced a higher proportion of smaller parenchymal and perivascular amyloid aggregates and in areas where plaques are not generally found in the non-treated animal, such as in the corpus callosum (Figure 5A), whereas cow seeds seem to trigger the formation of larger plaques. The differences in the seeding competency and the profile of aggregates induced suggest that Aβ aggregates present in human and cattle brain may represent different arrangements or “conformational strains” of Aβ seeds.

## Discussion

The prion-like induction and spreading of misfolded protein aggregates implicated in several protein misfolding disorders is a recently recognized process with potentially important implications to understand the etiology and progression of these diseases and the development of novel strategies for therapeutic intervention (Prusiner, 2012; Soto, 2012; Walker and Jucker, 2015). The prion principle of disease transmission posits that a misfolded protein aggregate is able to transfer biological information by converting the normal form of the protein into more of the abnormal, disease-associated form. This process initiates the progressive generation of misfolded protein aggregates that spread to other areas of the brain and accumulate over time to produce brain damage and disease. The prion-like transmission of the pathological folding—operating at the molecular and cellular levels—is probably at the root of the spreading of damage throughout the brain that is characteristic of neurodegenerative diseases (Moreno-Gonzalez and Soto, 2011; Thal et al., 2014; Walker and Jucker, 2015). However, in prion diseases transmission also occurs among different individuals in which prions act as *bona fide* infectious agents to spread disease in animal or human populations. Moreover, in the case of prions the disease can be transmitted across animals from different species (Béringue et al., 2008), with the landmark case being the generation of a new human disease, termed variant Creutzfeldt-Jakob disease, produced by exposure to cattle affected by bovine spongiform encephalopathy (Collinge et al., 1996; Bruce et al., 1997; Scott et al., 1999). Evidence gathered over the past 10 years has demonstrated that both protein aggregates implicated in AD (Aβ and tau) can propagate under experimental conditions as prions (Prusiner, 2012; Soto, 2012; Morales et al., 2015a,b; Walker and Jucker, 2015).

The main goal of our study was to investigate whether cattle present AD-like lesions in the brain, and if these pathological features display prion-like seeding activity. For this purpose, we characterized the brains from many cows at different ages for the presence of amyloid deposits and subsequently the ability of those aggregates to induce amyloid pathology in a mouse model of amyloid deposition. The results of this study show that some old cows spontaneously develop amyloid deposits in their brain. Interestingly, these deposits were similar to those present in human cases of AD. Out of the 63 cattle brain samples analyzed, nine of them (14.3%) displayed typical Aβ aggregates in the analyzed areas. Therefore, appearance of amyloid deposits in cattle brain appears at a frequency comparable to the prevalence of AD pathological abnormalities in humans, since it is estimated that AD affects about 6% of people 65 years and older, and the incidence doubles every 5 years. Of course, many more elderly non-demented people display amyloid pathology in their brains, a condition that is usually considered as pre-clinical or prodromal AD (Tagliavini et al., 1988; Price et al., 2009).

Cattle brains were also analyzed for hyperphosphorylated tau (ptau) and tangle formation. We found that only three specimens had elevated levels of ptau using AT8 antibody. These brains were not used as inoculum for the intracerebral inoculation study to specifically evaluate the effect of Aβ aggregates. We cannot exclude that brains could also contain other types of amyloid seeds including tau oligomers that we were unable to detect. Although tau aggregates can modulate Aβ toxicity, it has been reported that they are not able to induce Aβ aggregation (Bloom, 2014; Nisbet et al., 2015). This, together with the use of APP mice, allows evaluating the seeding capability of Aβ using complete brain homogenates, excluding the effect of potential other seeds present in the inoculum.

Although behavioral or cognitive abnormalities were not analyzed in cows, the amount of Aβ deposits observed parallels pre-clinical AD. Importantly, brain extracts from old cows containing Aβ aggregates were able to significantly increase amyloid deposition when injected intra-cerebrally into a double transgenic mouse model of AD amyloidosis. Though the total level of pathological induction was lower than the one produced by inoculation of AD brain homogenates, the cow material showed a higher promotion activity than human tissue when the rate of induction was corrected by the amount of Aβ aggregates injected. This surprising result suggests that cattle aggregates are better seeds than their human counterparts. The most likely explanation for this result is that cattle deposits are smaller, less compact and contain smaller amounts of other components compared to human aggregates, which presumably remain deposited in the brain for much longer periods of time. This interpretation is supported by our previous observations showing that brain tissue from people affected by mild cognitive impairment (MCI), which is considered a pre-clinical form of AD, produced significantly higher Aβ induction than tissue from established AD patients (Duran-Aniotz et al., 2013). Moreover, Jucker and colleagues have shown that small, soluble Aβ oligomers are better seeds *in vivo* than large fibrillar aggregates (Langer et al., 2011). We have recently published data demonstrating that AD patients displaying different amyloid pathology induce different pathological features in the same transgenic models used in this study (Duran-Aniotz et al., 2021). In that line, the distinctive pathology observed between cattle and human AD patients may be responsible of their dissimilar seeding activity. It could well be that these differences are due to the fact that cattle develop different strain(s) of Aβ, which are able to accelerate aggregation with smaller amounts of the original seeds or more efficiently. A more in-depth study would be needed to determine the presence of a different Aβ strain in cattle.

The prion-like transmission of Aβ aggregates has been extensively reported in animal models and likely plays an important role in the progressive spreading of pathological abnormalities throughout the brain (Moreno-Gonzalez and Soto, 2011; Thal et al., 2014; Walker and Jucker, 2015). Nevertheless, whether this phenomenon ever operates in the inter-individual transmission of disease pathology in humans remains highly debatable. Recent studies have provided evidence for the induction of Aβ aggregation in people receiving human pituitary-derived growth hormone (Jaunmuktane et al., 2015; Ritchie et al., 2017). However, when the risk of AD development, and not only amyloid pathology, was studied no evidence was found for disease transmission (Irwin et al., 2013). The findings of our current study suggest that Aβ aggregates present in the brains of old cattle are competent to seed amyloid deposition *in vivo*. This induction has also been observed with other protein aggregates such as AA amyloid (Rising et al., 2021). However, the potential transmission of Aβ cattle-derived seeds to humans is unlikely, considering that repeated oral administration of AD brain extracts to susceptible mice failed to accelerate pathological features (Morales et al., 2021). The results presented in this manuscript suggest that aged cattle are susceptible to develop pathological features similar to AD, and that misfolded Aβ present in their brain is seeding competent.

## Data Availability Statement

The raw data supporting the conclusions of this article will be made available by the authors on reasonable request.

## Ethics Statement

The studies involving human participants were reviewed and approved by Institutional Review Board, The University of Texas Health Science Center at Houston. Informed consent was obtained by the National Disease Research Interchange (NDRI). The animal study was reviewed and approved by Committee of Animal Use for Research, The University of Texas Health Science Center at Houston.

## Author Contributions

IM-G designed the experiments, participated in animal manipulation, performed the histological, biochemical, image, and statistical analyses, prepared the figures, and wrote the manuscript. GEIII participated in animal manipulation and performed the histological analysis. RM participated in animal manipulation and participated in the experimental design. CD-A performed histological, image, and statistical analysis. GEJr participated in histological analysis. MM and MP provided cattle samples and performed their initial neuropathological analysis. CS supervised the entire project, designed the research plan, and wrote the manuscript. All authors read and approved the final manuscript.

## Funding

This work was partially funded by the Alzheimer's Association (NIRP-12-257323 to IM-G, AARGD-18-566576 to RM), the Spanish Ministry of Science and Innovation PID2019-107090RA-I00 and Ramon y Cajal Program RYC-2017-21879 (IM-G), the National Institutes of Health RF1AG072491 (RM) and 3P01AI077774-09S1 to CS, and an award from the Mitchell Foundation (CS) 2018-AARG-591107, ANID/FONDEF ID20I10152, ANID/FONDECYT 1210622, and Anillo ACT210096 to CD-A.

## Conflict of Interest

The authors declare that the research was conducted in the absence of any commercial or financial relationships that could be construed as a potential conflict of interest.

## Publisher's Note

All claims expressed in this article are solely those of the authors and do not necessarily represent those of their affiliated organizations, or those of the publisher, the editors and the reviewers. Any product that may be evaluated in this article, or claim that may be made by its manufacturer, is not guaranteed or endorsed by the publisher.
