# Supplementary figures and images for: Aged Cattle Brain Displays Alzheimer's Disease-Like Pathology and Promotes Brain Amyloidosis in a Transgenic Animal Model
Source: Front Aging Neurosci. 2022 Jan 31;13:815361. doi: 10.3389/fnagi.2021.815361 (PMC8841674; doi:10.3389/fnagi.2021.815361)

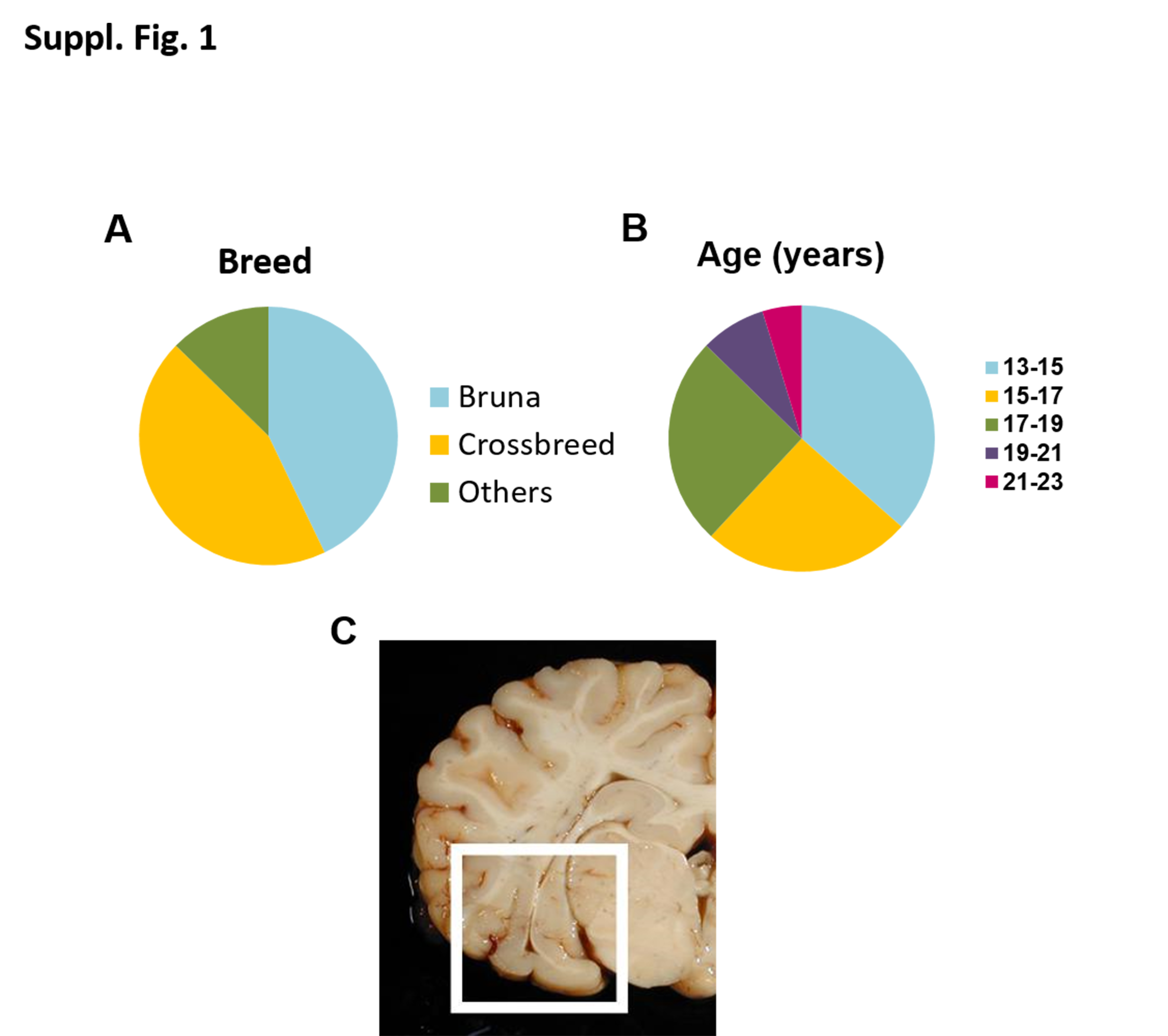

Supplement: Supplementary file 1 [file Image_1.TIF]
